# Sparse force-bearing bridges between neighboring synaptic vesicles

**DOI:** 10.1007/s00429-019-01966-x

**Published:** 2019-10-30

**Authors:** John F. Wesseling, Sébastien Phan, Eric A. Bushong, Léa Siksou, Serge Marty, Isabel Pérez-Otaño, Mark Ellisman

**Affiliations:** 1 Instituto de Neurociencias, CSIC-UMH, San Juan de Alicante, Spain; 2 Departmento de Neurociencias (CIMA), Universidad de Navarra, Pamplona, Spain; 3 National Center for Microscopy and Imaging Research, University of California, San Diego, CA USA; 4 Institut de Biologie de l’ENS (IBENS), École Normale Supérieure, INSERM, CNRS, PSL Research University, Paris, France; 5 Global Research and Development, Teva Pharmaceutical Industries Ltd, Netanya, Israel; 6 Institut du Cerveau et de la Moelle épinière, INSERM U1127, CNRS UMR7225, Université Pierre et Marie Curie, Sorbonne Universités, Paris, France; 7 National Center for Microscopy and Imaging Research and Department of Neuroscience, University of California, San Diego, CA USA

**Keywords:** RRP, Reserve, Supply-rate depression, Presynaptic, Tomography

## Abstract

Most vesicles in the interior of synaptic terminals are clustered in clouds close to active zone regions of the plasma membrane where exocytosis occurs. Electron-dense structures, termed bridges, have been reported between a small minority of pairs of neighboring vesicles within the clouds. Synapsin proteins have been implicated previously, but the existence of the bridges as stable structures in vivo has been questioned. Here we use electron tomography to show that the bridges are present but less frequent in synapsin knockouts compared to wildtype. An analysis of distances between neighbors in wildtype tomograms indicated that the bridges are strong enough to resist centrifugal forces likely induced by fixation with aldehydes. The results confirm that the bridges are stable structures and that synapsin proteins are involved in formation or stabilization.

**Electronic supplementary material:**

The online version of this article (10.1007/s00429-019-01966-x) contains supplementary material, which is available to authorized users.

## Introduction

A key step in chemical synaptic transmission is exocytosis of neurotransmitter stored within vesicles in presynaptic terminals. Exocytosis occurs at the active zone region of the plasma membrane, but space is only available for docking a few of the hundreds within resting terminals. The non-docked vesicles are held in clouds in the interior (Pieribone et al. 1995; Schikorski and Stevens 1997; Rey et al. 2015). The current concept is that some of the vesicles within the clouds occupy reserve pools that can be recruited to the active zone when needed (Denker and Rizzoli 2010).

Electron-dense bridges have been detected between some pairs of vesicles within the clouds (Landis et al. 1988; Hirokawa et al. 1989). Although initially hypothesized to underlie cloud formation, each vesicle was bridged to an average of only 1.5 of the approximately 7 contiguous neighbors in Schaffer collateral terminals, and a different kind of mechanism for cloud formation has been proposed (Siksou et al. 2007; Milovanovic et al. 2018). Based on this, the idea that bridges are stable structures in vivo—as opposed to artifacts of tissue preparation—and the relevance for function, have been questioned (Wang and Kaeser 2018).

**Fig. 1:**
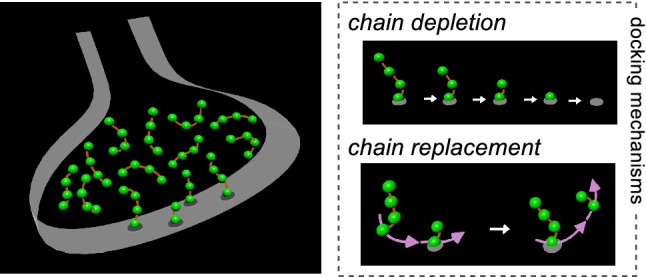
Model of cloud where synaptic vesicles are tethered together into short chains. This scenario was proposed in Gabriel et al. (2011) to explain simplifying mathematical constraints that emerged from electrophysiological studies of rate-limiting steps in synaptic vesicle cycling at Schaffer collateral synapses. Vesicles throughout the terminal are linked together in short chains. Non-docked vesicles attached to docked vesicles serve as an autonomous reserve pool that can be expended/depleted during heavy use. Docked vesicles are replaced stochastically at the slow rate of 1/min with a vesicle at the start of a full chain that was previously not docked

However, our own working model of rate-limiting steps in vesicle recruitment to the active zone at Schaffer collateral terminals does ascribe a critical function to the bridges that is very much in-line with the sparse distribution, and even predicts the precise value of 1.5 per vesicle (Gabriel et al. 2011). The idea is that vesicles throughout the terminals are connected into short chains consisting of only four vesicles (Fig. 1).

**Fig. 2:**
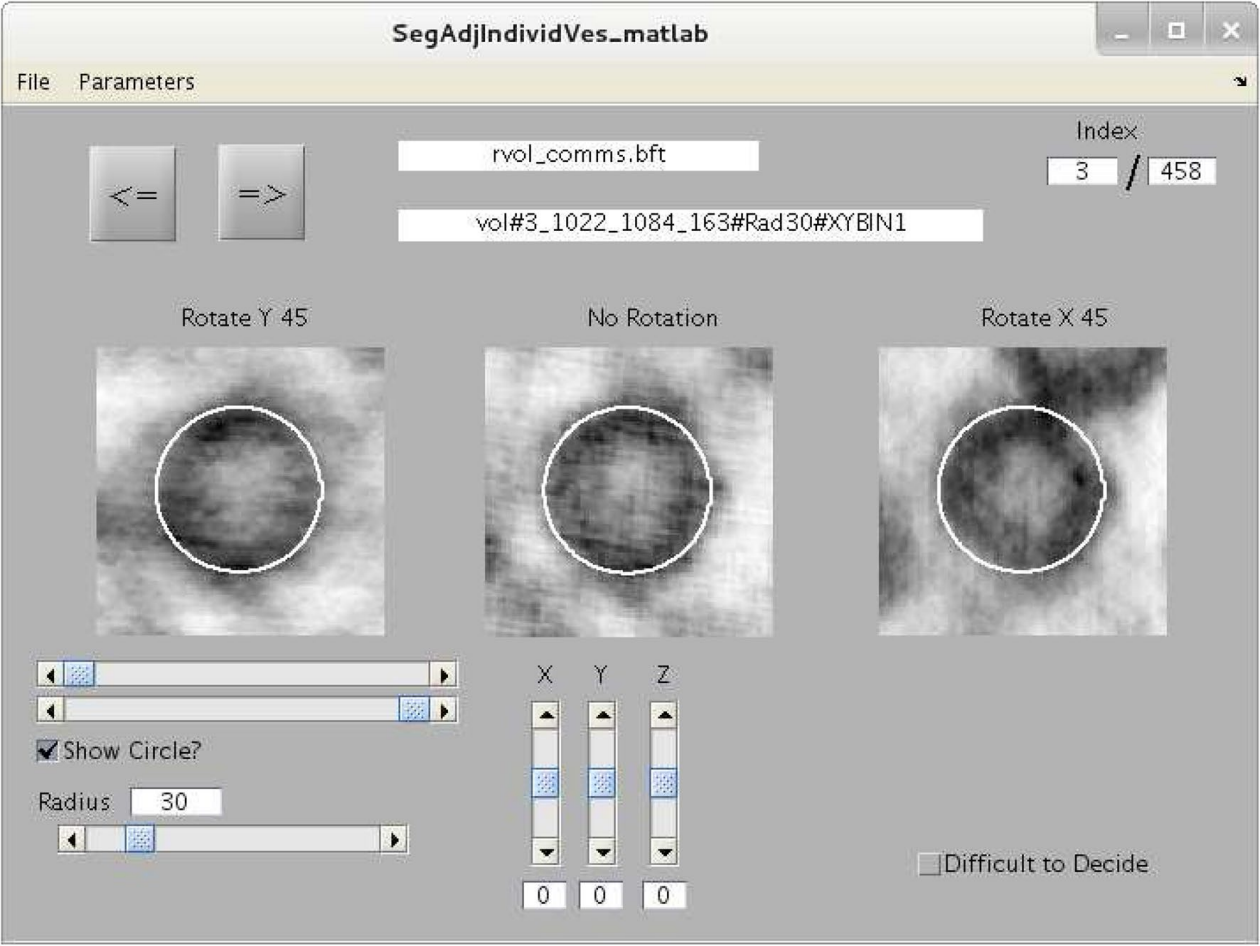
Graphical user interface used for fine tuning estimates of vesicle center and radius. As a first step in the analysis, rough estimates for the position and size of each vesicle within each tomogram were obtained by modeling each vesicle with a sphere using the 3dmod computer program of the IMOD suite of software (Kremer et al. 1996). A separate mini-tomogram containing each vesicle was then extracted from the whole tomogram. The three images in the user interface are all the means of virtual slices from the same mini-tomogram spanning the estimated center point (i.e., spanning a total of 0.5 median vesicle diameters). The center image is the mean of virtual slices parallel to the horizontal plane without rotating the mini-tomogram, the leftmost image is after rotating the mini-tomogram $45^{\circ }$  about the *y*-axis, and the rightmost image is the mean after rotating about the *x*-axis. The white circle is the perimeter of the sphere calculated from the center and radius estimates and will match all three images when the estimates are correct. The horizontal sliders directly below the leftmost image determine contrast. The bottom-most horizontal slider allows the observer to adjust the radius estimate. The vertical sliders allow the user to adjust the estimated location of the center point

The chains were included in the model to explain functional results that are not related to the discussion about whether or not the bridges are involved in cloud formation. Specifically, the chains can explain evidence against the idea at the core of most other models that mass action plays a role in the timing of vesicle recruitment to the active zone (Stevens and Wesseling 1999; Garcia-Perez et al. 2008; Gabriel et al. 2011); the chains would dock to the active zone as a unit, endowing each release site with an autonomous reserve pool that would be replenished, stochastically, as an all-or-none event. We were, therefore, interested in determining if the bridges are stable enough to play a role in vivo as a consistency check for the working model.

The molecular composition of the bridges is not known, but synapsin proteins have long been hypothesized to play a role (Landis et al. 1988; Benfenati et al. 1989; Hirokawa et al. 1989; Hilfiker et al. 1999). If our working model is correct, the chains would continue to be present in knockout synapses missing synapsin 1 and 2, but would have to be shorter, and the number of links per vesicle would be reduced (Gabriel et al. 2011). A previous electron tomography study demonstrated that at least some of the bridges remain in synapsin knockouts, but did not resolve whether the number per vesicle was altered (Siksou et al. 2007). We were, therefore, additionally interested in determining if the number is reduced in synapsin knockout synapses.

Standard segmentation techniques for comparing the bridges in tomograms from wildtype (WT) and synapsin knockout synapses can be complicated by the fact that the density of vesicles within clouds is substantially lower in knockout synapses (Li et al. 1995; Rosahl et al. 1995; Gitler et al. 2004; Siksou et al. 2007). However, we have now developed a new method for quantifying the bridges that avoids this complication.

We detected a substantial reduction in the fraction of pairs of neighboring vesicles that are connected by a bridge in knockouts after fixation with aldehydes, along with other indications that the bridges are real structures in vivo, and not artifacts of tissue preparation. A follow-on analysis of the distribution of distances between neighbors indicated that the bridges are strong enough to resist centrifugal forces likely induced by vesicle shrinkage caused by the fixation procedure. Taken together, the results indicate that the bridges play a physical role in tethering vesicles together in vivo and confirm the involvement of synapsin proteins in formation or stabilization.

## Methods

### Tissue preparation

Age-matched 14- to 21-day old WT and synapsin DKO mice were first anesthetized with ${20}\,{\upmu } \hbox {l}/\hbox {g}$ of a cocktail containing 3% ketamine and 0.08% xylazine. Brains were then fixed by transcardial perfusion with a ringer solution containing 2 UI/ml heparin and 0.02% xylocaine followed by a fixation solution containing 2% paraformaldehyde, 2.5% glutaraldehyde, and 0.15 M cacodylate (Giepmans et al. 2005). Fixed brains were stored at ${4}\,^\circ \hbox {C}$ for up to 4 days in the same solution before sectioning at ${100}\,{\upmu } \hbox {m}$, high-pressure freezing, freeze substitution with tannic acid, then osmium tetroxide in acetone, and infiltration with Durcupan resin and polymerization as described in Sosinsky et al. (2008). ${300}{\hbox { nm}}$ sections were collected on 200 hexagonal mesh grids coated with 0.5% formvar and then stained with 2% aqueous uranyl acetate (${20}{\hbox { min}}$) or Sato’s lead (${5}{\hbox { min}}$) (Sato 1968), or both in sequence as indicated. The grids were then coated with either 5 or ${10}{\hbox { nm}}$ colloidal gold particles as fiducial markers, and electron microscopy images of classically defined asymmetric synapses in stratum radiatum of the hippocampus were acquired at 300 keV on an FEI Titan microscope at 37,000 $\times$ (Group 1 in Fig. 5) or 29,000 $\times$ (Groups 2 and 3) magnification.

### Image acquisition

Two tilt series were recorded for each specimen from at least $-\,50^{\circ }$ to $+\,50^{\circ }$ at 1$^{\circ }$ increments; the specimen was rotated 90$^{\circ }$ in the horizontal plane between the first and second tilt series (Mastronarde 1997).

### Tomogram reconstruction

The etomo program of the IMOD suite (Kremer et al. 1996) was used for fiducial alignment and standard data manipulations prior to back projection. Back projection reconstruction was then performed using the TxBR software package (Lawrence et al. 2006).

### Segmentation

Center location and radius of vesicles was estimated by a two-step procedure. First, rough estimates were obtained by modeling each vesicle with a sphere using the 3dmod computer program of the IMOD suite. Then, a separate mini-tomogram containing each vesicle was extracted from the whole tomogram, and estimates were fine tuned with the graphical user interface depicted in Fig. 2.

### Experimental design and statistical analyses

Experimental design is described throughout the “Results” section. Statistical analyses were performed as indicated throughout the “Results” and figure legends.

**Fig. 3:**
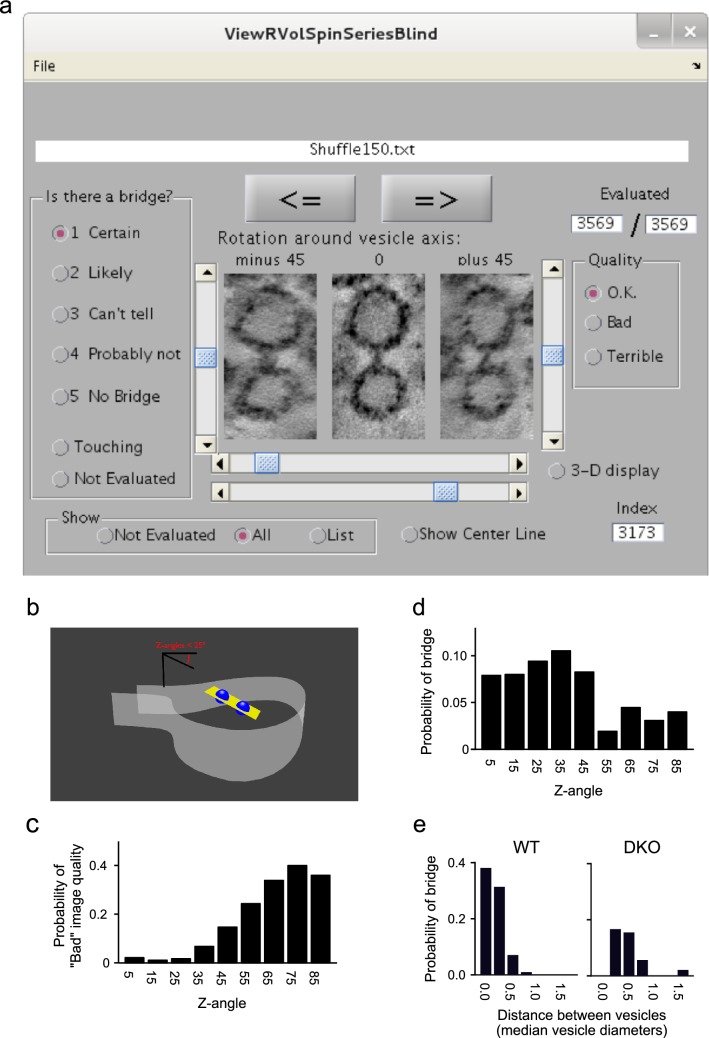
Method for evaluating the presence or absence of bridges between vesicles. **a** Screenshot of graphical user interface for evaluating presence or absence of bridges. Mini-tomograms for each vesicle pair were rotated so that the centers of both vesicles were within the horizontal plane. Images of vesicle pairs were then presented in a random order simultaneously from three angles: not rotated; and rotated $\pm {45}^{\circ }$ about the central axis common to both vesicles. When the “3-D display” radio button is selected, the scroll bar to the right allows the user to scroll through the individual virtual sections. Otherwise, the mean images of all virtual sections between 0.25 median vesicle diameters above and below the central plane are displayed. The lower scroll bars below the images control contrast. **b**–**e** Initial survey of 13 WT and 15 DKO tomograms for vesicle pairs separated up to 1.5 vesicle diameters. **b***Z*-angle for a pair of vesicles is defined as the angle between the axis passing through the centers of both vesicles and the plane that is horizontal to the tissue slice. **c** Fraction of WT vesicle pairs for which images were evaluated as qualitatively “Bad” or “Terrible” vs *Z*-angle. **d** Probability of detecting a bridge vs *Z*-angle in WT synapses. Scores of 1 (“Certain”) and 2 (“Likely”) were counted as bridges. **e** Probability of detecting a bridge vs distance between the vesicles for *Z*-angles $\le \,{25}^{\circ }$ (640 pairs for WT, 374 for DKO). Distance units are median vesicle diameters, which were calculated across all vesicles for each tomogram independently

## Results

We employed a combination of chemical and rapid high-pressure freezing techniques to fix brains of age-matched WT and synapsin 1 and 2 double knockout mice (DKO); mice were between 14 and 21 days old to match the range used in previous functional studies (Garcia-Perez et al. 2008; Gabriel et al. 2011). The fixation procedure has previously been shown to preserve labile tissue ultrastructure for electron tomography at a level that is comparable to rapid high-pressure freezing alone (Sosinsky et al. 2008), and allowed us to avoid artifacts related to dissecting and freezing living tissue, and to avoid shipping live animals between laboratories. Classically defined asymmetric synapses in stratum radiatum of the hippocampus were then imaged with an electron microscope from multiple tilt angles along two orthogonal axes and tomograms with at least ${6.4}\,{\AA }$ resolution were generated as described in “Methods” (view the supplementary Video for an example). Bridges between some vesicles were obvious, particularly in WT tomograms (Fig. 3a). See below for multiple types of evidence that the bridges were real structures and not artifacts of chemical fixation or freezing.

### Detection procedure

Vesicles within synapsin DKO terminals were less crowded together than vesicles in WT terminals owing to fewer vesicles overall as reported previously (Rosahl et al. 1995). The difference was readily apparent while browsing through the tomograms using the 3dmod computer program of the IMOD suite (Kremer et al. 1996), precluding unbiased blind comparisons at the level of whole tomograms. However, we did not use automatic machine detection algorithms because of the concern that false negatives or false positives might occlude relevant differences.

Instead, we first estimated the center location and radius of individual synaptic vesicles (see “Methods”). No difference was detected in the size of vesicles at WT vs synapsin DKO synapses; the mean, across tomograms, of the median vesicle diameter for each was $37.6\pm 0.5\,\hbox {nm}$ for WT and $39.6\pm 0.5\,\hbox {nm}$ for synapsin DKO. Median vesicle diameter of each tomogram was used as our standard measure of length for comparisons across tomograms throughout the study.

We then used computer programs to: (1) automatically extract a separate miniature tomogram for each vesicle pair; and (2) present images from each miniature tomogram in a random order to an observer who was asked to score for the presence or absence of a bridge on a five point scale (Fig. 3a). The observer was additionally given the option of indicating that the vesicles appeared to be touching, which would make detecting a bridge impossible, and if images were perceived to be of low quality.

We conducted an initial survey on a subset of vesicle pairs separated by up to 1.5 median vesicle diameters from 13 WT and 15 DKO tomograms. A maximum of 150 vesicle pairs from each tomogram was included in the analysis. The pairs were chosen at random, irrespective of distance to landmarks such as the plasma membrane or active zone (but see below). Pairs where neither vesicle was at least 1.5 vesicle diameters from artificial edges of the vesicle cluster created by tissue sectioning were excluded. The full analysis consisted of 1631pairs for WT and 931 for DKO because some tomograms contained fewer than 150 such pairs. Tomograms containing fewer than 50 pairs (all synapsin DKO) were excluded.

Scores of 1 (“Certain”) and 2 (“Likely”) were counted as bridges. The survey revealed degradation in image quality (Fig. 3b, c), and information content (Fig. 3d), for vesicle pairs that were oriented more than 45$^{\circ }$  from the horizontal plane, likely resulting from the so-called missing wedge effect inherent to tomograms reconstructed from axial tilt series (Frank 2006). The survey additionally revealed that almost all bridges were between vesicles separated by 0.5 median vesicle diameters or less (Fig. 3e).

### Fewer bridges between neighbors in synapsin DKO synapses

**Fig. 4:**
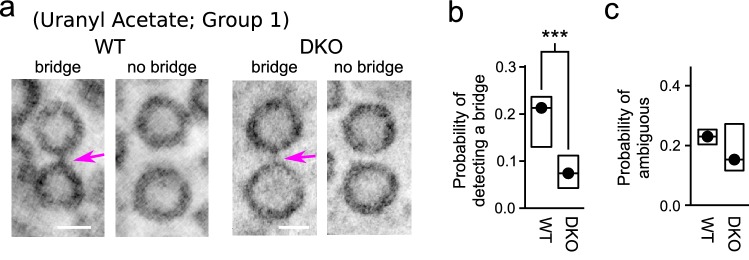
Lower probability of detecting a bridge between neighboring vesicles in synapsin DKO synapses. **a** Images of pairs of vesicles with and without a bridge. Magenta arrows indicate bridges. Scale bars are 30 nm. Image thickness was 8.3 nm (i.e., average of 13 virtual sections with a voxel spacing of ${6.4}\,{\AA }$). Post-fix staining for these examples was with uranyl acetate, but no lead, which is referred to as “Group 1” below. **b** Probability of detecting a bridge per pair of neighbors vs genotype. Values were estimated individually for each tomogram by dividing the number of bridges detected by the number of pairs that were analyzed. Circles are the median values across tomograms, boxes delineate the middle two quartiles, ***$p<0.001$ (Kolmogorov–Smirnov; $n=13$ tomograms for WT, 15 for DKO). **c** No difference in scores of “3 Can’t tell”

**Fig. 5:**
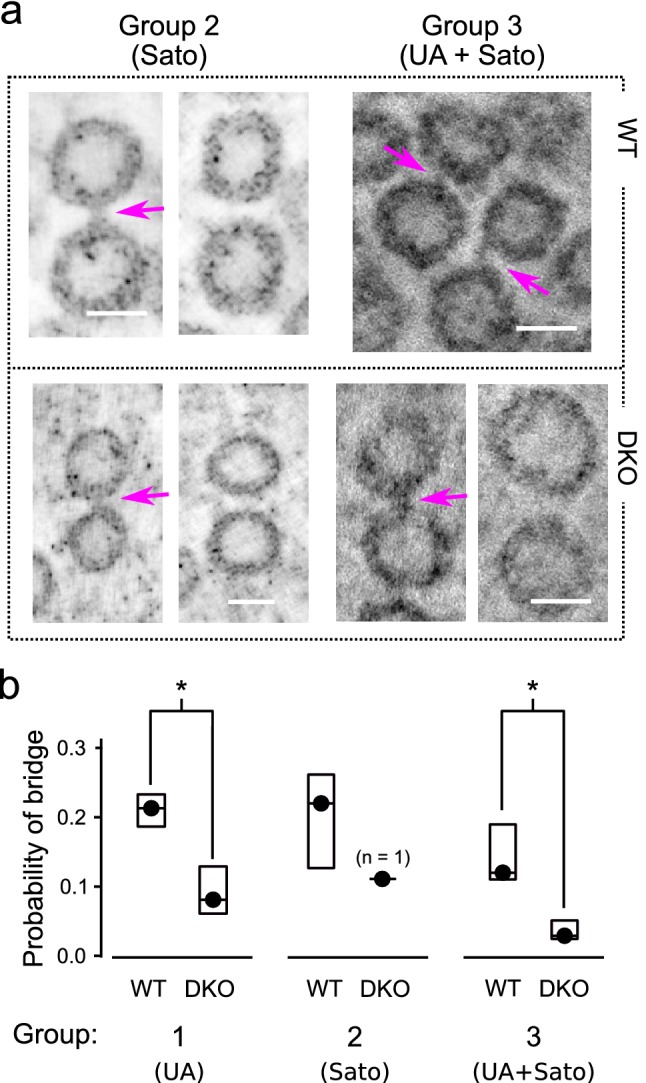
Similar differences between WT and synapsin DKO across several post-fixation staining protocols. **a** Examples of vesicle pairs with and without bridges after two distinct post-fixation protocols. See Fig. 4a for Group 1 examples. Magenta arrows indicate bridges. Scale bar is 30 nm. **b** Quantification identical to Fig. 4b for each protocol ($n=5$ and 10, respectively, for WT and DKO tomograms for Group 1; 5 and 1 for Group 2; and 3 and 4 for Group 3; *$p<0.05$, Kolmogorov–Smirnov)

The results in Fig. 3e suggest that equivalently spaced vesicles are less likely to be linked together by a bridge in synapsin DKO compared to in WT synapses, in line with the model proposed in Gabriel et al. (2011). For higher resolution information, we next conducted a second random survey, again containing a maximum of 150 vesicle pairs from each tomogram, but this time the pairs were drawn from the subset that were oriented within 25$^{\circ }$  of the horizontal plane and had separation distances between vesicles of 0.5 median vesicle diameters or less. For this second survey, the full analysis consisted of 1950 pairs for WT and 1673 for DKO; more pairs were available per tomogram despite the additional constraints on separation distance and angle because pairs were included where neither vesicle was less than 0.5 vesicle diameters from artificial edges of the vesicle cluster, compared to the 1.5 vesicle diameters excluded in the first survey. We found that the probability of detecting a bridge between neighboring vesicles was more than twofold lower for synapsin DKO synapses (Fig. 4a, b), whereas no difference was detected between WT and synapsin DKO in the fraction of pairs judged to be ambiguous (Fig. 4c). Similar results were obtained for specimens post-fixed with Uranyl Acetate with or without Sato’s lead staining (Groups 1 and 3 in Fig. 5). Sato’s staining without Uranyl Acetate yielded similar results for WT tissue, but only a single tomogram of a synapsin DKO synapse surpassed the lower limit of 50 pairs meeting criteria (Group 2 in Fig. 5) precluding any test for statistical significance.

The mean distance between vesicles within the pairs was slightly greater for synapsin DKO synapses (i.e., even though all were within the 0.5 median vesicle diameter limit established by the criteria). However, the lower probability of detecting a bridge between neighboring synapsin DKO vesicles could not have been simply because the vesicles were farther apart because the relative difference between WT and synapsin DKO was even greater when the analysis was restricted to the shortest distances. For example, bridges were only detected in 3 of 42 synapsin DKO pairs (7.1%) for separation distances of less than 0.1 median vesicle diameters, but 41 of 112 WT pairs (36.6%; $p<0.001, {\chi }^{2}$ with Yate’s correction).

**Fig. 6:**
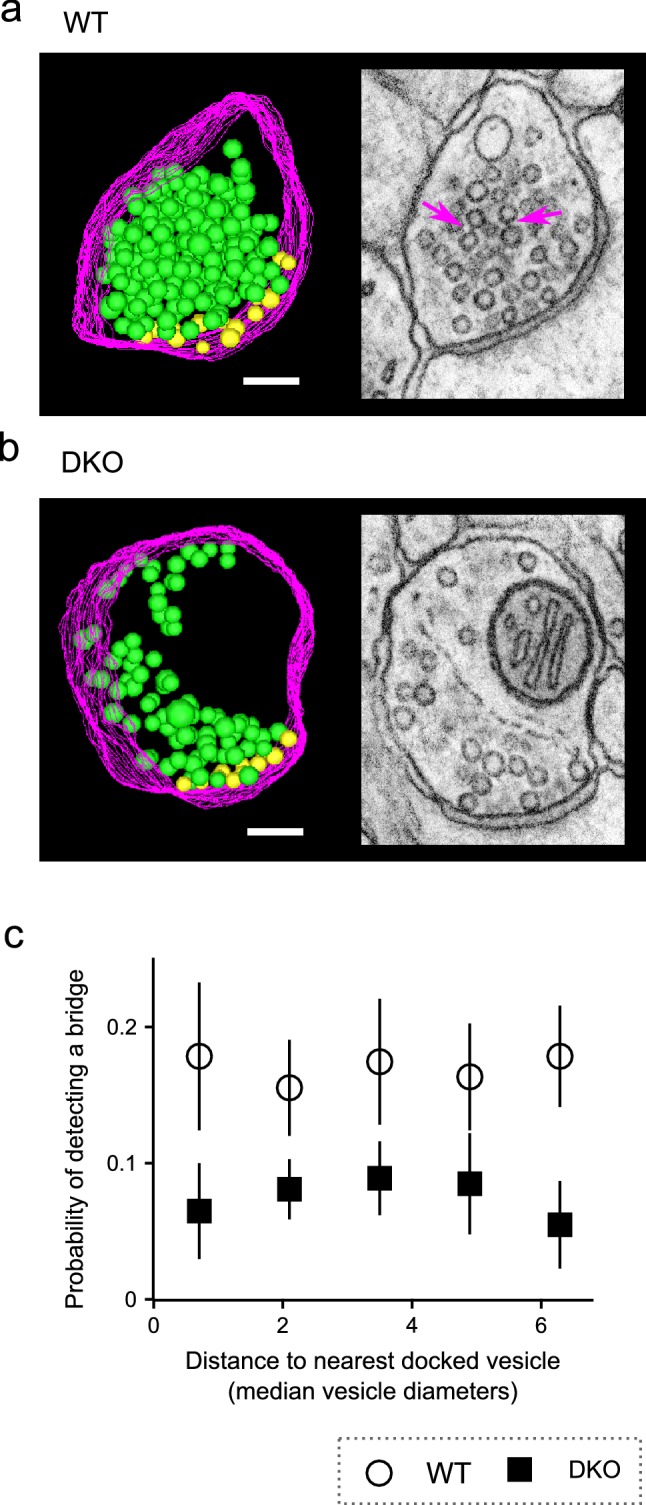
No decrease in bridges near active zone. **a**, **b** Left panels are two-dimensional snapshots of three-dimensional models used to determine vesicle locations. Yellow spheres are docked vesicles, green are non-docked, magenta lines are plasma membrane. Right panels are examples of image slices (${6.4}\,{{\AA }}$ thick) showing docked and non docked vesicles. Magenta arrows indicate bridges. Models and images correspond to the same tomogram, but the tilt angles are slightly different to better illustrate the docked vesicles. Scale bars are 100 nm and pertain to both the model and corresponding image slice. **a** WT. **b** DKO. **c** Fractions of pairs with a bridge vs distance between the geometric center of the vesicle pairs and the center of the closest docked vesicle. Same data set as Figs. 4 and 5; every tomogram had at least two docked vesicle because only synapses where the synaptic cleft was visible were selected for imaging. Mean number of docked vesicles/tomogram was ${13.7}\,\pm \,{1.8}$ for WT and ${10.3}\,\pm \,{1.5}$ for DKO (not significant, Kolmogorov–Smirnov). Probability of detecting a bridge was calculated for each range of distances for each tomogram separately and then averaged across tomograms; $n=13$ tomograms for WT and $\ge \,{14}$ for DKO instead of the 15 in previous figures because no pairs were present farther than 4 median vesicle diameters from the active zone for one of the DKO tomograms

These results provide strong evidence that most of the bridges are real structures and not artifacts of tissue preparation that would pertain equally to WT and synapsin DKO tissue; this is the first of multiple types of evidence against fixation artifacts noted above. No differences were detected in the probability of finding a bridge between neighbors located proximal vs distal to the active zone for either genotype (Fig. 6). However, the analysis was specifically designed to detect bridges between pairs of vesicles; the increased amount of synapsin seen at locations distal to the active zone using immunochemical techniques in previous studies might reflect soluble synapsin in a liquid phase or bridges to elements of the cytoskeleton which would not have been detected here (Evergren et al. 2007).

**Fig. 7:**
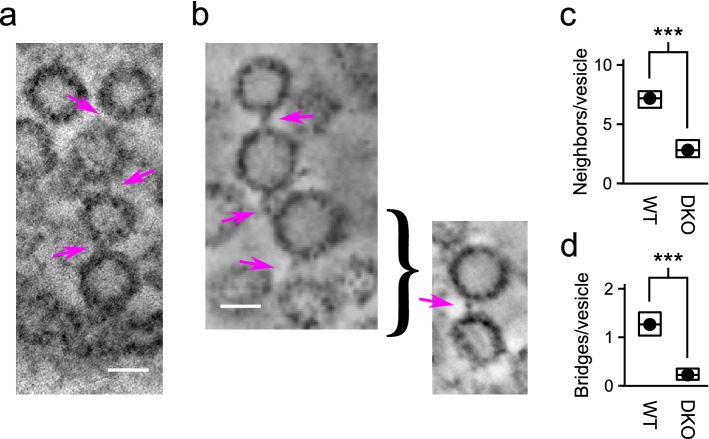
WT chains of four vesicles. **a** Examples of chain where one of the vesicles was docked. **b** Example where only two bridges are visible in any single plane, but the third can be seen from a different angle. Magenta arrows indicate bridges and scale bars are 30 nm. **c** Mean number of neighbors within 0.5 median vesicle diameters of each vesicle. The lower value for DKO implies lower density of vesicles in space, which is in-line with previous reports. **d** Bridges per vesicle estimated by multiplying the probability of a bridge in Fig. 4b by the number of neighbors per vesicle in **c**

### Quantification of WT bridges is consistent with new model

As noted in the “Introduction”, our working model predicts that vesicles in WT synapses are connected into short chains of four vesicles (Gabriel et al. 2011). The technical limitations documented above caused by the missing wedge phenomenon (i.e., Fig. 3c–e) precluded identification of full chains in most cases. However, we did find clear examples of chains with four vesicles while browsing through the full tomograms using the 3dmod computer program of the IMOD suite (Kremer et al. 1996) (Fig. 7a, b). In addition, information about the number of neighbors per vesicle was available from the initial step of the analysis where the center location and diameter of vesicles throughout the tomograms were estimated (Fig. 7c). This information could be combined with the probability of detecting a bridge in Fig. 4b to extrapolate the mean number of bridges from each vesicle to neighbors (Fig. 7d). The resulting value of ${1.3}\pm {0.1}$ for WT tomograms was close to the value estimated in a previous study where tomograms were acquired from tissue that was rapidly frozen without first fixing with aldehydes (Siksou et al. 2007). The close agreement provides quantitative support for the conclusion in Sosinsky et al. (2008) that the combination of chemical fixation followed some time later by rapid high-pressure freezing can preserve the ultrastructure of labile tissue. In addition, the values are in line with the prediction of our working model that vesicles are connected together in short chains consisting of four vesicles; if the model is correct, the mean number *n* would be $n=\frac{(2\cdot r)-2}{r}$, where *r* is the number of vesicles in a chain and *n* would be 1.5 for WT because $r=4$.

### Synapsin DKO bridges are less detectable

However, we detected only $0.24\pm 0.04$ bridges per synapsin DKO vesicle (Fig. 7d), which is less than predicted by the model; i.e., chains in synapsin DKO synapses were predicted to contain $r=2.6$ vesicles, on average, in which case each vesicle would be connected to 1.2 others. The result does not support the model per se, but does not rule it out either because some bridges in synapsin DKO synapses may have escaped detection owing to lower contrast compared to WT bridges. And indeed, bridges were more difficult to detect in synapsin DKO tomograms; only 1.8% (3 of 165) of all bridges were scored as 1’s (as opposed to 2’s), whereas 4.8% (18 of 372) of WT bridges were scored as 1’s ($p<0.05, {\chi }^{2}$ with Yate’s correction). Or, some of the bridges may have broken during fixation (see below). In any case, synapsin DKO vesicles had ${3.2}\pm {0.4}$ (s.e.m.) neighbors on average (Fig. 7c), which is safely more than the minimum of 1.2 required of the model.

**Fig. 8:**
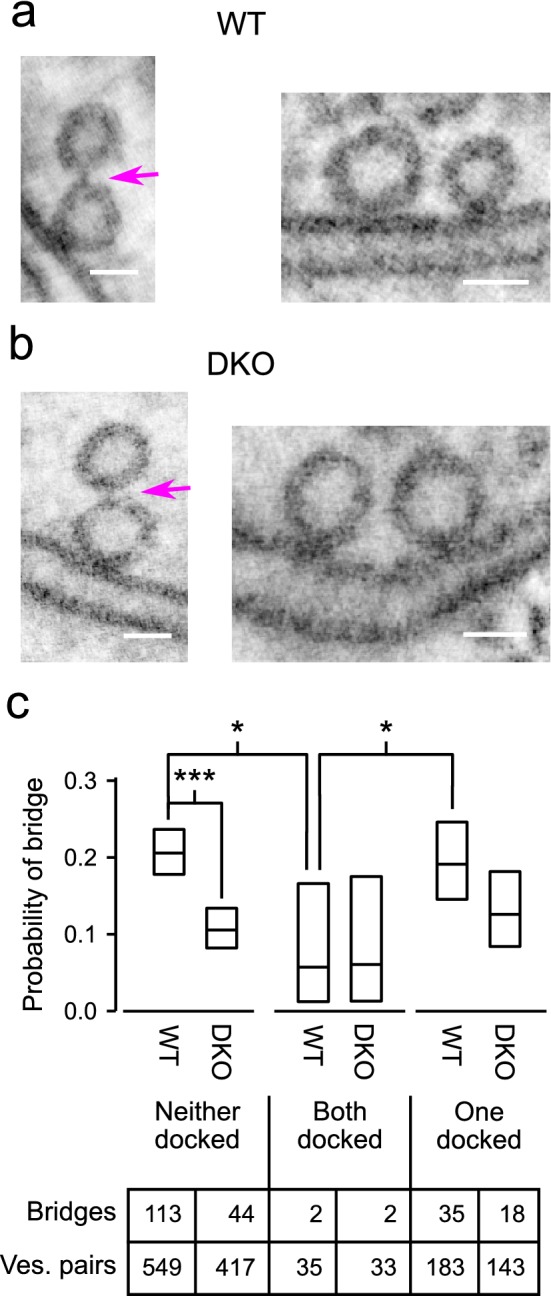
Comparison of bridges between non-docked and docked vesicles. The analysis included all vesicle pairs where at least one of the two vesicles was docked, along with a randomly selected subset of vesicle pairs where neither vesicle was docked (*Z*-angle was $\le \,{25}^{\circ }$ for all pairs). **a**, **b** Images of WT and DKO pairs where one of the vesicles is docked and is bridged to the other in the cytoplasm (left panels), and where both vesicles are docked (right panels). Scale bars are 30 nm, thickness of virtual section was 8.3 nm. Magenta arrows indicate bridges. **c** Quantification: horizontal lines within boxes are fraction of pairs judged to be connected by a bridge across the entire analysis, which is a different type of quantification than used above (see “Results”). Boxes delineate the 90% confidence interval for binomial proportions using the Wilson score interval with correction for continuity (Newcombe 1998); ***$p<0.001$, *$p<0.05$; both using ${\chi }^{2}$ with Yate’s correction. Raw values are listed in the table

### No evidence for lateral bridges between docked vesicles

Next, an additional analysis focused on bridges between pairs of vesicles of which one or both were docked (Fig. 8). The probability of finding a bridge between pairs when both were docked was lower compared to when neither was docked (first vs third box in Fig. 8c) or compared to when only one of the pair was docked (fifth vs third box). For this analysis, we compared binomial proportions calculated directly from the data sets as a whole instead of the median values calculated individually for each tomogram used above. The two types of comparison yielded similar results (compare leftmost 2 bars in Fig. 8c to Fig. 4b), but comparison of binomial proportions was more appropriate in this case because of the near absence of bridges between pairs of docked vesicles (i.e., only 68 docked pairs met the criteria established above, and of those, we detected only 4 putative bridges in all 28 tomograms). Indeed, the 4 may well have been false positives, which would be in-line with our working model because docked vesicles are predicted to be connected to vesicles within the interior, not laterally to other docked vesicles.

### Spatial inhomogeneity within vesicle clouds

Finally, an analysis of vesicle locations within the clouds confirmed that the bridges are strong enough to bear force.

**Fig. 9:**
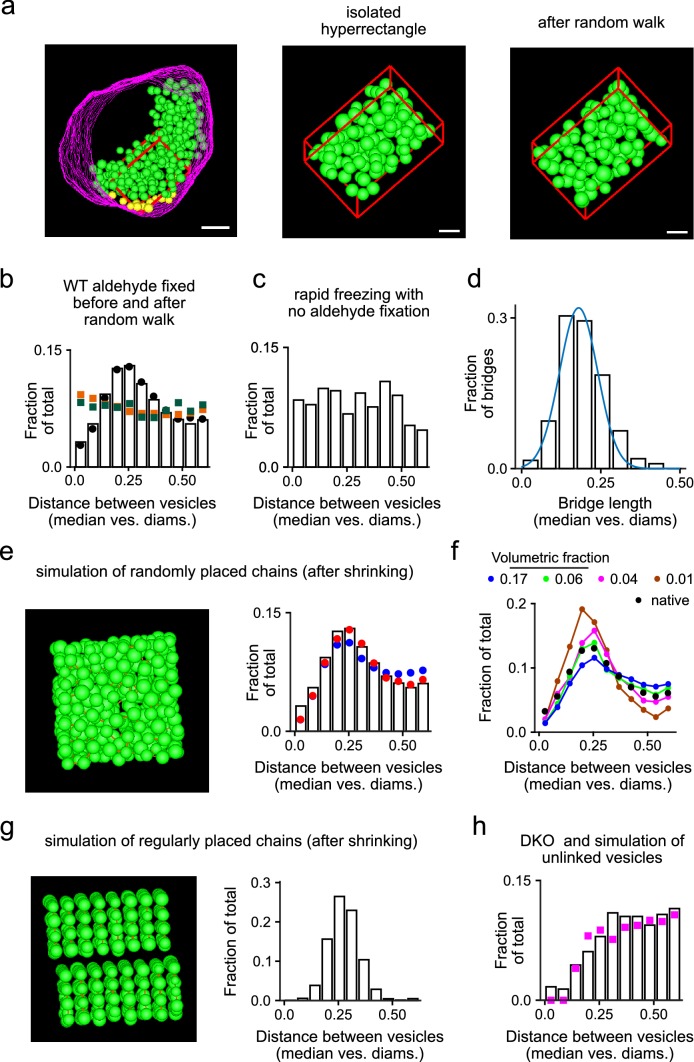
Inhomogeneities in spatial distribution are predicted by short chains connected by force-bearing bridges. **a** Example of hyperrectangular subset of vesicles before and after simulating a random walk in space. **b** Bars are fraction of WT vesicle pairs vs separation distance between vesicles in each pair in hyperrectangles before the random walk. Black circles are the same measurement for the entire WT data set, which includes vesicles inside and outside the hyperrectangles. Green squares represent pairs in hyperrectangles after the random walk. Orange squares represent pairs from simulations of cloud formation where vesicles that were not connected into chains were placed at random locations in a cube. **c** Analogous histogram for tomograms from Siksou et al. (2009) where tissue was frozen rapidly without chemical fixation ($n=316$ pairs from seven tomograms containing a mean of ${36.7}\,\pm \,{6.3}$ vesicles/tomogram; one of eight tomograms was excluded owing to too few pairs). **d** Histogram of measurements of bridge lengths from the WT data set used in simulations. The blue line is a normal distribution with mean of 0.18 and standard deviation of 0.06. **e** Left panel is a snapshot of a simulated cloud where vesicles connected into chains of four were placed at random locations in a cube. Each vesicle was shrunk by 13% after cloud formation to simulate the shrinkage that likely occurs during aldehyde fixation. Bridges are represented by the sparsely distributed brown rods. The bars in the right panel are identical to in **b**; blue circles are the analysis of simulations matching the snapshot to the left; red circles are after adding a small amount of order as described in “Results”. **f** Analogous histogram for simulations where vesicles were connected into chains of four for a variety of volumetric fractions. The blue circles are the same as in **e**; the simulations of lower volumetric fractions were then generated by eliminating randomly selected chains. Native data are the same as in **b**. **g** Left panel is a snapshot of a simulated cloud where vesicles connected into chains of four were placed in an ordered rather than random formation; volumetric fraction was 0.17. Right panel is histogram of separation distances between vesicles in each pair. **h** Analogous histogram for DKO tomograms; compare to bars in **b**. Magenta squares are from simulations of cloud formation where vesicles were not connected into chains, followed by shrinkage by 12%; volumetric fraction was 0.07 for both tomograms and simulation

That is, non-docked vesicles were not randomly distributed in space as would be expected of independently mobile constituents of a liquid phase. This can be seen by comparing histograms of the distances between neighbors to the analogous histograms calculated from: (1) the same tomograms after simulating diffusion of the individual vesicles using a random walk algorithm as depicted in Fig. 9a (compare bars to green squares in Fig. 9b); or, (2) simulations of cloud formation generated by randomly placing spheres in an initially empty space (compare bars to orange squares in Fig. 9b).

For this analysis, simulations were simplified by limiting the spatial bounds to hyperrectangles rather than by approximating the irregular borders of real vesicle clouds. Random walks were applied to hyperrectangular subsets from the interiors of vesicle clouds (e.g., red box in Fig. 9a), and cloud formation was simulated within initially empty $300 \times 300 \times 300$ nm cubes. The simplification was appropriate because limiting spatial bounds of simulations to hyperrectangles did not alter the distribution of distances between neighbors (compare black circles to bars in Fig. 9b).

Randomly distributed vesicles touched in some cases (non-zero values of leftmost green and orange points in Fig. 9b), but neighbors in actual tomograms rarely came close (leftmost bar in Fig. 9b is close to zero), indicating that some mechanism kept vesicles apart. In principle, the absence of touching could be caused by mutually repellent forces between vesicles, but was more likely to have occurred during tissue fixation because aldehydes are known to cause organelles to shrink—likely owing to extrusion of water—which would open spaces between vesicles (Murk et al. 2003); a similar mechanism may account for aldehyde induced flattening of vesicles in Gray’s type 2 synapses (Gray 1969; Rapisardi and Lipsenthal 1984; Korogod et al. 2015). And indeed, vesicles did appear to touch in tomograms of tissue that was fixed by rapid freezing without aldehyde fixation (leftmost bar of Fig. 9c); and, the diameters of aldehyde fixed vesicles are consistently less than when unfixed tissue is frozen rapidly. We estimated a reduction in diameter of 10–13%; the 13% value was obtained by comparing the mean of vesicle diameters here (39.6 nm) to 45.6 nm in Imig et al. (2014), whereas the 10% value emerged from the modeling study documented next.

Simulating shrinkage or mutual repulsion after cloud formation did prevent vesicles from touching, but could not be tweaked to reproduce the peak in the WT distribution occurring at about 0.25 median vesicle diameters that can be seen in Fig. 9b. Instead, the peak aligned closely with that of the distribution of lengths of the bridges between vesicles (Fig. 9d), suggesting that the bridges restricted mobility. And indeed, simulating cloud formation by placing chains of vesicles—rather than individuals—at random locations in an initially empty space, and subsequently reducing the diameter of each vesicle ${13}{\%}$ to simulate shrinkage, did generate distributions with peaks that matched actual tomograms (blue circles in Fig. 9e).

A perfect match was only obtained when the density of vesicles in the simulation was lower than the density in actual tomograms. That is, density was quantified as the *volumetric fraction* by dividing the sum of the volumes of all vesicles in the hyperrectangles by the volume of the hyperrectangles. Volumetric fraction in WT tomograms was ${0.17}\pm {0.01}$, but simulations only matched actual measurements when the volumetric fraction was lowered to 0.06 (compare black and green circles in Fig. 9f).

However, the remaining mismatch at a volumetric fraction of 0.17 could be eliminated by introducing a small amount of order to the placement of chains, for example by combining the random placement with the regular placement depicted in Fig. 9g at a ratio of 85 to 15 (red circles in Fig. 9e). In this case, the best match was obtained when vesicle shrinkage was 10% rather than 13%.

A key point is that the distribution of distances between neighbors could only be reproduced if bridges were modeled as rigid structures that were strong enough to drag vesicles closer together to compensate for the decreases in diameter. Also relevant, bridge lengths in simulations were chosen by randomly sampling from the distribution of bridge lengths measured in tomograms, whereas the distribution of distances between neighbors could not be reproduced if bridge lengths were instead drawn from wider or narrower distributions, or from distributions with substantially different means. Moreover, the distribution of distances between neighbors within DKO synapses, where bridges were fivefold less frequent (Fig. 7d), could be fit by simulations where individual vesicles were placed at random locations and then shrunk by between 10 and ${13}{\%}$ (Fig. 9h).

Taken together, these results suggest strongly that the bridges in WT synapses provide force for holding vesicles together. If our working model is correct, the placement of the chains would not be completely random in space, consistent with an additional level of organization (Siksou et al. 2007; Fernández-Busnadiego et al. 2010; Schrod et al. 2018), or simply crowding/compaction by a still unidentified mechanism.

### Tethered pairs overlap after reversing shrinkage

The conclusion that bridges provide physical force for holding vesicles together was strengthened further by the observation that neighboring vesicles connected by bridges were closer together than neighbors that were not connected (${0.19}\pm {0.004}$ median vesicle diameters versus ${0.31}\pm {0.005}$). And, simulating the reverse of shrinkage by increasing the diameter of each vesicle by 15%—i.e., $\tfrac{{45.6}\,{\rm nm}}{{39.6}\,{\rm nm}}$—often caused neighbors to overlap if they were connected by a bridge (${23}{\%}$; 71 of 306), but almost never if they were clearly not connected (3%; 16 of 496). The difference was statistically significant ($p<1{\text{E}}-5$; ${\chi }^{2}$), and was not simply because the pairs without a bridge were farther apart because the fraction of overlaps only rose to 11% (14 of 132; $p<{0.003}$) for pairs separated by up to only 0.23 vesicle diameters; i.e., for which the mean separation distance (${0.19} \pm {0.002}$) matched the mean for neighbors that were connected by bridges.

## Discussion

Here we report: (1) a new method for detecting bridges connecting neighboring vesicles in electron microscopy tomograms; that we have used to (2) demonstrate a reduction in synapsin knockouts in the probability that any two neighbors are connected. Previous doubts about the relevance of the bridges for synapse structure and function are countered by multiple independent indications that the bridges are real structures that can bear force rather than artifacts of sample preparation.

The bridges have long been suspected of containing synapsin proteins (Landis et al. 1988; Benfenati et al. 1989; Hirokawa et al. 1989). However, our results do not support the long-standing synapsin hypothesis where vesicles would be heavily interconnected in a dense network (rev. in Hilfiker et al. 1999). Instead, individual vesicles were only connected to an average of 1.3 of 7 neighbors (i.e., $\sim$ 20%).

Nor do our results prove that synapsin proteins are constituents of the bridges. Instead, synapsins might be involved in bridge creation or stabilization by a more indirect mechanism (Orenbuch et al. 2012a). And indeed, we found that at least some bridges continued to be present in synapsin DKO terminals. The sparse distribution of bridges and the observation that at least some remain in synapsin knockouts was reported previously (Siksou et al. 2007), but, to our knowledge, the present study is the first to demonstrate that the probability of finding a bridge between neighbors is altered in synapsin knockouts.

Our results do not argue against the recent proposal that clouds of vesicles are held together by a liquid phase of quickly associating and dissociating synapsin molecules (Milovanovic et al. 2018). If so, individual vesicles would not be free to move around independently, but individual chains of vesicles might be. However, our results are equally consistent with the idea that short chains of vesicles are fixed within a larger cytoskeletal structure (Siksou et al. 2007; Fernández-Busnadiego et al. 2010; Schrod et al. 2018), in which case motion would instead be directed by motor proteins.

### Model where vesicles are linked into short chains

Our initial motivation was to conduct a consistency check for our working model of rate-limiting steps in synaptic vesicle trafficking (Fig. 1), which was originally proposed to explain purely functional results that are incompatible with the premise of conventional models that the timing of vesicle recruitment to a readily releasable pool is influenced by mass action of vesicles in reserve pools (Stevens and Wesseling 1999; Garcia-Perez et al. 2008; Gabriel et al. 2011). The present results provide qualitative support for the new model by confirming that, at least in Schaffer collateral synaptic terminals: (1) vesicles throughout the clouds are tethered together; (2) synapsins are involved; and, (3) docked vesicles are tethered to vesicles in the interior.

The results are in line with the quantitative prediction that vesicles are each tethered to an average of 1.5 others in WT Schaffer collateral terminals. However, quantitative aspects of the results must be taken with caution because of an unknown number of false negatives. And, indeed, the probability of finding a bridge between a given pair of vesicles in synapsin DKO terminals was substantially less than predicted, which might simply be because bridges in DKO terminals were more difficult to detect, or were weak enough to be broken during fixation.

Finally, Fig. 1 depicts vesicles arranged in linear chains, but the missing wedge phenomenon documented in Fig. 3b–d prevented us from ruling out branching arrangements. In any case, the working model was generated to explain functional results that suggested that vesicles in WT Schaffer collateral synapses are held together in groups of 4, which would be compatible with both branched and linear arrangements. A key point, however, is that, while compatible with the working model, the results do not rule out other possible arrangements such as much longer chains.

### New method

A key methodological complication was the well-established observation that synaptic terminals from synapsin knockouts contain fewer vesicles than WT and that the spatial distribution is less dense (Li et al. 1995; Rosahl et al. 1995; Gitler et al. 2004; Siksou et al. 2007). The lower density is noticeable when tomograms are viewed as a whole, precluding blind comparisons using standard segmentation techniques. An alternative strategy for avoiding biases produced by human judgments would be to develop a computer program for detecting the bridges automatically (e.g. Fernández-Busnadiego et al. 2010). However, automatic detection can be less sensitive than the human eye, meaning that the algorithms may produce more false positives, more false negatives, or both, which could have occluded key results. In addition, automatic algorithms sometimes require human input—such as setting threshold levels—which can also introduce judgment biases.

Instead, we partitioned the initial tomograms into mini tomograms containing slices through the center of neighboring pairs of vesicles. At this scale, it was no longer possible to distinguish individual WT and synapsin DKO samples. We then presented an extended, randomized series of the mini tomograms to an observer who evaluated the space between each pair of vesicles for the presence or absence of a bridge (see Fig. 3a). The procedure allowed us to take advantage of the facility of human vision for pattern detection to produce quantitative estimates of the probability of finding bridges between pairs of neighboring vesicles that were not compromised by biases caused by preconceptions of the observer.

Why eliminating synapsin decreases the number of vesicles is not known. One possibility would be that vesicles that are not incorporated into the chains hypothesized by our working model are targeted for degradation simply because they are free floating (Li et al. 1995; Orenbuch et al. 2012b), but other mechanisms continue to be possible as well (Sun et al. 2006; Gaffield and Betz 2007).

Note that our analysis was designed specifically to evaluate whether the bridges between vesicles seen in WT synapses are reduced in number after knocking out synapsins. A different method would be needed to evaluate whether synapsins are additionally involved in tethering vesicles to cytoskeletal elements (Evergren et al. 2007).

### Controls for fixation artifacts

The tissue used for the present study was prepared first by chemical fixation followed by rapid freezing up to several days later, which allowed us to avoid shipping live animals between laboratories, and to study synapses that were more deeply embedded in tissue than allowed by rapid freezing alone (Korogod et al. 2015 reported ice damage at depths of greater than 10–20 ${\upmu } \hbox {m}$). We are aware that non-native structures produced by chemical fixation may have complicated the interpretation of some previous electron microscopic studies and that omitting this step before freezing can reduce the impact of such factors in some cases (Wolosewick and Porter 1979). However, we are confident that most of the bridges detected in our tomograms were native structures, and not the product of fixation, because: (1) the combination of chemical fixation with freezing used here has previously been shown to preserve labile tissue ultrastructure for electron tomography at a level that is comparable to rapid high-pressure freezing alone (Sosinsky et al. 2008); (2) the probability of finding a bridge between neighboring vesicles is greatly reduced in synapsin DKO synapses; (3) if present, fixation artifacts would have additionally been found between neighboring pairs of docked vesicles, but this was almost never seen; and (4) an analysis of local inhomogeneities in the positions of vesicles within clouds indicated that the bridges were strong enough to resist centrifugal forces likely produced by vesicular shrinkage during the chemical fixation stage. In any case, quantitatively similar numbers of bridges between vesicles were seen in tomograms from the same brain region when unfixed tissue was frozen rapidly (Siksou et al. 2007).

### Synapsin 3

The synapsin family contains three genes, but we analyzed synapsin 1 and 2 double knockouts rather than triple knockouts so that results could be compared directly to our previous functional study (Gabriel et al. 2011). However, we would not expect triple knockout synapses to be substantially different because synapsin 3 is not thought to be expressed in Schaffer collateral terminals during the 14–21 day range analyzed here (Hosaka and Südhof 1998; Ferreira et al. 2000), and because synapsin 3 appears to play a functional role that is qualitatively different compared to synapsins 1 and 2 (Feng et al. 2002). In addition, the previous tomography study has already demonstrated the continued presence of bridges between vesicles in synapses from the triple knockouts (Siksou et al. 2007).

## Electronic supplementary material

Below is the link to the electronic supplementary material.

Supplementary material 1 (PDF 3387 kb)
